# Effects of immunoglobulin plus prednisolone in reducing coronary artery lesions in patients with Kawasaki disease: study protocol for a phase III multicenter, open-label, blinded-endpoints randomized controlled trial

**DOI:** 10.1186/s13063-021-05807-3

**Published:** 2021-12-11

**Authors:** Si-Yuan Lin, Lan He, Li-Ping Xie, Yin Wang, Yi-Xiang Lin, Yin-Yin Cao, Wei-Li Yan, Fang Liu, Guo-Ying Huang

**Affiliations:** 1 Heart Center, Children’s Hospital of Fudan University, Shanghai, 201102 China; 2 Clinical Trial Unit, Children’s Hospital of Fudan University, Shanghai, 201102 China

**Keywords:** Kawasaki disease, Primary treatment, Corticosteroid, Coronary artery lesions

## Abstract

**Background:**

Kawasaki disease (KD) is an acute systemic vasculitis of unclear etiology that mainly affects infants and young children. Strategies to reduce the incidence and severity of coronary artery lesions (CALs), the determinant factor in the long-term prognosis of KD, are currently a focus of studies on KD. Corticosteroids, preferred in the treatment of the majority of vasculitides, are controversial in the treatment of acute KD. In this trial, we will evaluate whether the addition of prednisolone to standard intravenous immunoglobulin (IVIG) plus aspirin therapy can reduce the occurrence of CAL in Chinese patients with KD.

**Methods:**

This is a multicenter, prospective, open-label, randomized controlled trial, which is expected to be conducted in more than 20 hospitals in China and aims to assess the efficacy and safety of IVIG + prednisolone treatment versus standard treatment. Patients with KD who fulfill the inclusion and exclusion criteria will be recruited and randomized (1:1) to receive either a large dose of IVIG (2 g/kg over 12–24 h with a maximum dose of 60 g) + aspirin 30 mg/kg/d or IVIG (2 g/kg over 12–24 h) + aspirin 30 mg/kg/d + prednisolone (2 mg/kg/d with a maximum dose of 60 mg tapered over 15 days after normalization of C-reactive protein concentration). The primary outcome will be the occurrence of CAL at 1 month of illness. The follow-up duration for each participant will be set as 1 year. Patients and treating physicians will be unmasked to group allocation.

**Discussion:**

This will be the first multicenter randomized controlled trial to evaluate the efficacy of IVIG + aspirin + prednisolone in Chinese pediatric patients with KD, which may provide high-level evidence for improving the initial treatment for acute KD.

**Trial registration:**

ClinicalTrials.govNCT04078568. Registered on 16 August 2018.

**Supplementary Information:**

The online version contains supplementary material available at 10.1186/s13063-021-05807-3.

## Background

Kawasaki disease (KD) is an acute systemic vasculitis of unclear etiology that mainly affects children under 5 years of age [1]. KD has been reported worldwide and is more prevalent in Asia, especially in Japan, where the incidence rate was 330.2 and 309.0 per 100,000 children < 5 years of age in 2015 and 2016, respectively [2]. In Shanghai, China, the annual incidence rate of KD per 100,000 children <5 years is also increasing, ranging from 68.8 in 2013 to 104.6 in 2017 [3].

The development of coronary artery lesions (CALs) is the most important complication and the main determinant of the long-term prognosis of KD. CAL can contribute to coronary artery aneurysms, occlusion, myocardial ischemia, myocardial infarction, and even death, making it a major cause of acquired heart disease in developed countries [4]. Although large doses of intravenous immunoglobulin (IVIG) have been demonstrated to reduce the development of CALs [5, 6], the incidence of medium to giant coronary aneurysms remains as high as 3.4% in China [3]. Thus, focus has been placed on the means to provide aggressive treatment with superior efficacy in reducing the occurrence of CAL.

Corticosteroids, preferred in the treatment of the majority of vasculitides, are controversial in the acute treatment of KD. An early meta-analysis conducted by Wooditch et al. [7] indicated that using corticosteroids in aspirin-containing regimens for primary treatment of KD could reduce the incidence of coronary aneurysms, which was maintained when IVIG was added to the treatment regimen. In 2007, a randomized trial assessing the efficacy of the addition of a single pulsed dose of intravenous methylprednisolone to conventional IVIG therapy did not provide support for this conclusion [8]. Findings from another randomized trial in 2012 evaluating the efficacy of immunoglobulin plus prednisolone for preventing CAL showed benefits for children with severe KD in Japan based on the Kobayashi scoring system [9]. Similarly, a meta-analysis conducted by Zhu et al. revealed that corticosteroids plus conventional treatment may reduce the incidence of CAL in patients with KD, and Chen et al. further demonstrated the importance of timing in another meta-analysis [10, 11]. All results suggest that the therapy time, regimen, and target group require careful consideration in the use of corticosteroids for KD treatment. As a vasculitis, using corticosteroids in the early therapy of KD may benefit not only those who are at high risk of IVIG resistance but also all patients with KD.

In light of these findings, we have designed this trial, a phase III, multicenter, prospective, open-label, randomized controlled trial aimed at assessing the efficacy of the addition of prednisolone to conventional initial treatment in children with acute KD. This study will be conducted in more than 20 hospitals within the Chinese Kawasaki Disease Collaboration Network.

## Methods

### Objectives

1. To determine whether IVIG + aspirin + prednisolone combination therapy as the primary treatment is superior to conventional IVIG + aspirin treatment in reducing the occurrence of CAL in patients with KD.
2. To optimize the initial treatment regimen for acute KD.

### Trial design

**Fig. 1:**
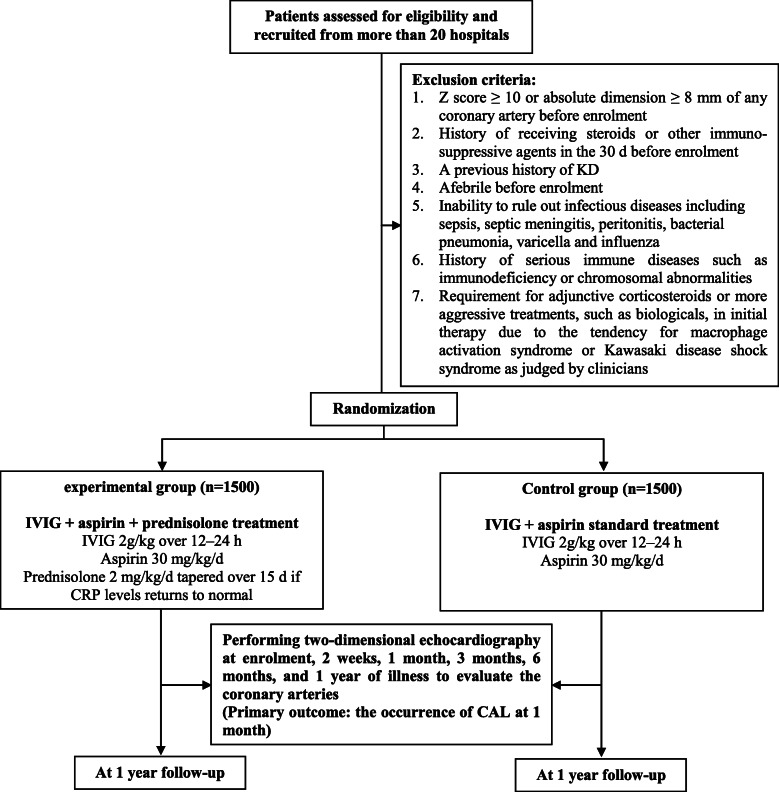
Diagram of the study design. CAL, coronary artery lesion; CRP, C-reactive protein; IVIG, intravenous immunoglobulin; KD, Kawasaki disease

**Fig. 2:**
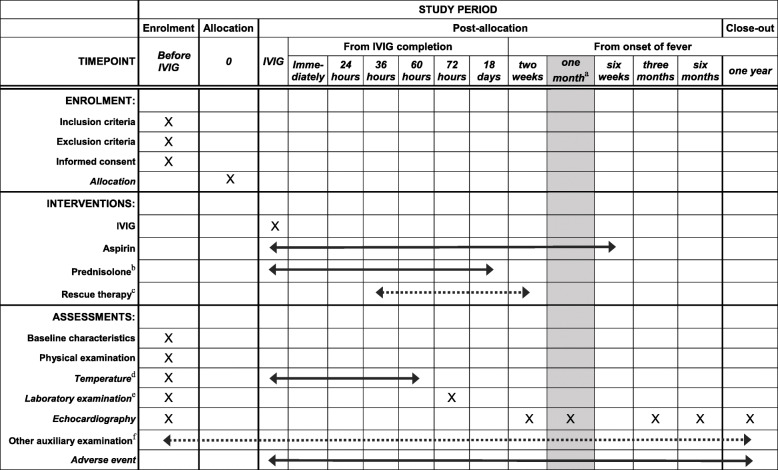
Schedule of enrolment, intervention, and assessment. **a** Gray shading shows the primary endpoint. **b** It is inferred experientially that CRP levels may become normal 3 days after IVIG completion when the patient switches from intravenous methylprednisolone to oral prednisolone tapered over 15 days. **c** Patients will accept rescue therapy if they exhibit IVIG resistance. **d** Axillary temperature (or rectal temperature) will be measured every 6 h from IVIG treatment. The time point, temperature, and treatment will be recorded if fever occurs between two measurements. When IVIG starts and body temperature becomes normal it will be recorded. **e** Laboratory examination includes CRP, ESR, Hct, Hb, ALB, SAA, prealbumin, ALT, AST, CK-MB, sodium, NT-proBNP, IL-2, IL-4, IL-6, IL-10, TNF-α, TB, troponin, D-dimer, lipid, and lipoprotein serum levels and WBC, NEUT, and PLT counts. CRP and routine blood tests will be measured every 3 days after completion of initial IVIG infusion until normal. The remaining indicators (except for ESR), if abnormal, will also be measured every 3 days after completion of initial IVIG infusion until normal. **f** Other auxiliary examination includes electrocardiogram, chest radiography, magnetic resonance angiography, and myocardial perfusion imaging. The patient will choose whether to accept the examinations based on the physical condition during the diagnostic and therapeutic period. ALB, serum albumin; ALT, alanine aminotransferase; AST, aspartate transaminase; CK-MB, creatine kinase-muscle/brain; CRP, C-reactive protein; ESR, erythrocyte sedimentation rate; Hb, hemoglobin; Hct, hematocrit; IL-2, interleukin-2; IL-4, interleukin-4; IL-6, interleukin-6; IL-10, interleukin-10; IVIG, intravenous immunoglobulin; NEUT, neutrophil; NT-proBNP, N-terminal pro-B-type natriuretic peptide, PLT, platelet; SAA, serum amyloid A; TB, total bilirubin; TNF-α, tumor necrosis factor-alpha; WBC, white blood cell

This is a multicenter, prospective, open-label, randomized controlled trial that will be carried out in more than 20 hospitals with adequate experience in diagnosing and treating KD. With written informed consent, patients who meet the eligibility criteria will be enrolled and randomly assigned in a 1:1 ratio to the control (receiving 2 g/kg IVIG over 12–24 h and 30 mg/kg/d aspirin) or experimental group (receiving 2 g/kg/d IVIG, 30 mg/kg/d aspirin, and an additional 2 mg/kg/d prednisolone). Assessments of coronary arteries will be conducted at baseline, 2 weeks, 1 month, 3 months, 6 months, and 1 year of illness. The primary outcome will be the occurrence of CAL at 1 month of illness. In addition, the duration of fever, changes in laboratory data, the percentage requiring rescue therapy, changes in *Z* scores, and occurrence of CAL at every time point of echocardiography during the study period will be compared between the two groups. All adverse events (AEs) will be reported. The study protocol is reported in accordance with the Standard Protocol Items: Recommendations for Clinical Interventional Trials (SPIRIT) 2013 statement (Additional File 1). A flow chart of the trial design is shown in Fig. 1. Figure 2 shows the study timeline according to the SPIRIT diagram.

The World Health Organization Trial Registration Data Set is included in the protocol to serve as a brief structured summary of the trial (Additional File 2).

### Eligibility criteria

Eligible patients refer to those meeting all inclusion criteria and none of the exclusion criteria listed below:

*Inclusion criteria:*

1. Meeting the diagnostic criteria for KD published by the American Heart Association (AHA) in 2017 [12], including complete KD (also known as typical or classic KD) and incomplete KD (also known as atypical KD)
2. Diagnosed within 10 days of onset (including the 10th day and the 1st day of onset defined as the first day of fever)
3. Not yet treated with IVIG
4. Age ≥ 1 month at the time of enrolment into the trial

*Exclusion criteria:*

1. *Z* score ≥ 10 or absolute dimension ≥ 8 mm of any coronary artery before enrolment
2. History of receiving steroids or other immunosuppressive agents 30 days before enrolment
3. A previous history of KD
4. Afebrile (axillary temperature lower than 37.5°C for at least 24 h) before enrolment
5. Inability to rule out infectious diseases, including sepsis, septic meningitis, peritonitis, bacterial pneumonia, varicella, and influenza
6. History of serious immune diseases, such as immunodeficiency, or chromosomal abnormalities
7. Requirement for adjunctive corticosteroids or more aggressive treatments, such as biologicals, in initial therapy due to the tendency for macrophage activation syndrome or Kawasaki disease shock syndrome as judged by clinicians

### Recruitment and informed consent

The study is expected to be conducted in more than 20 hospitals in China, distributed among at least 10 provinces and municipalities in the eastern, western, northern, southern, and central parts of China. Under the precondition of ensuring randomization, no specific number of subjects enrolled by each participating institute will be required.

In each institute, patients diagnosed with KD will be considered as the participants in this trial. The research physician will discuss information regarding the main aspects of the trial with the parent or guardian, as well as with the patients who are determined to have the ability to understand the trial and obtain written consent from the parent or guardian if the children with KD are eligible and the parent or guardian is willing to allow them to participate in the trial. In the case of patients over the age of 10, a signed informed consent from the patient will be needed in addition to that provided by the parent or guardian.

### Randomization: sequence generation and allocation concealment

According to the random seeds generated by the SAS software (version 9.4; SAS Institute, Cary, NC, USA), numbers from 1 to 6 were randomly generated by a computer for each block, and each number corresponded to one of six plans for allocating four participants of the block to two arms in a 1:1 ratio. Each allocation sequence was placed in small, opaque, and sealed envelopes numbered and marked in order from one to four and were enclosed in a larger, opaque, and sealed envelope marked with the block number. Randomization was stratified by study site. A randomization plan and concealed envelopes were prepared by an independent team of statisticians from the Clinical Trial Unit (CTU) of the Children’s Hospital of Fudan University.

Recruited patients will be randomly assigned in a 1:1 ratio to the control or experimental group through blocked randomization using the center as the stratified factor. After obtaining signed informed consent from the eligible participant, the trained physician will meet with the prespecified institutional nurse for the allocation plan for the very participant, as indicated by the corresponding envelope. Block envelopes and the four enclosed small envelopes will be required to be opened for enrolment and recruitment. The nurse will administer the allocation record form in front of the physician, and the form from each site will be monitored weekly using a data monitor from the central hospital.

### Blinding

Patients and physicians will not be masked to the assignment. Pediatric cardiologists who assess CAL using echocardiography will be blinded to the assignment.

### Interventions

Participants in the control group will receive IVIG 2 g/kg and oral aspirin 30 mg/kg per day (given over three doses) in the initial treatment. Participants in the experimental group will receive IVIG 2 g/kg, oral aspirin 30 mg/kg per day (given over three doses), and prednisolone 2 mg/kg per day in the initial treatment.

IVIG will be administered over 12–24 h with a maximum dose of 60 g. The dose of aspirin will be reduced to 3–5 mg/kg per day after three fever-free days and normalization of C-reactive protein (CRP) level, and its administration will continue for at least 6 weeks after the onset of KD. Patients in the experimental group will receive intravenous methylprednisolone 1.6 mg/kg per day, which is given in two doses (equal to prednisolone 2 mg/kg, and the maximum dose is 60 mg of prednisolone), then switched to oral prednisolone 2 mg/kg after three fever-free days. If CRP levels return to normal, the prednisolone dose will be tapered over 15 days in 5-day steps, from 2 mg/kg per day to 1 mg/kg per day to 0.5 mg/kg per day. During corticosteroid administration, 0.5 mg/kg of omeprazole will be administered daily for gastroprotection.

In both groups, patients resistant to initial IVIG therapy will receive rescue therapy, including a second dose of IVIG (2 g/kg), a high dose of methylprednisolone (10–30 mg/kg per day), infliximab (5 mg/kg), other immunosuppressive agents, a combination of two or more drugs, or even more aggressive treatment, such as plasmapheresis, depending on patient condition and physician experience. IVIG resistance is defined as recurrent or persistent fever (axillary temperature ≥ 37.5°C or rectal temperature ≥ 38°C) 36 h after completion of the initial IVIG infusion.

For patients developing CALs, the investigators will follow up for at least 3 years and provide recommendations for treatment and physical activity based on the guidelines “Recommendations for clinical management of Kawasaki disease with coronary artery lesions (2020 revision)” [13] during the follow-up period.

The schedule for the study data collection is described in Fig. 2.

### Assessment and outcome measures

The baseline characteristics of each patient will be collected, including sex, date of birth, height, weight, clinical manifestations, subtype of KD, days of fever before initial IVIG, echocardiographic findings at enrolment, a series of pre-IVIG laboratory tests, and the time at which initial IVIG is administered.

Two-dimensional echocardiography will be performed to evaluate the alternation in diameters of the coronary arteries at six time points: at enrolment (before initial IVIG treatment), 2 weeks of illness (± 2 days, before discharge), 1 month of illness (+ 5 days), 3 months of illness (± 5 days), 6 months of illness (± 5 days), and 12 months of illness (± 5 days). In some cases, such as for patients with severe CALs, echocardiography assessments will be more frequent and relevant results will also be recorded. The measurement for each patient’s echocardiogram will include the internal diameters of the left main coronary artery (LMCA), left anterior descending artery (LAD), left circumflex coronary artery (LCX), and proximal and middle segments of the right coronary artery (RCA). Echocardiography will be performed by pediatric echocardiographers at each participating center. Video recordings will be preserved and reevaluated by two other pediatric cardiologists for confirmation. The *Z* score of each coronary artery will be calculated based on height, weight, and measured diameter of the coronary artery [14].

### Primary outcome measurement

The primary outcome will be the occurrence of CAL at 1 month of illness. According to the guidelines published by the AHA in 2017, CAL is defined as Z ≥ 2 of any coronary arteries of the LMCA, LAD, LCX, and proximal and middle segments of the RCA and is stratified based on the maximal *Z* score (*Z*_max_) and diameters of all coronary arteries. Dilation only is defined as *Z*_max_ ≥ 2 to < 2.5; small aneurysms are defined as *Z*_max_ ≥ 2.5 to < 5; medium aneurysms are defined as *Z*_max_ ≥ 5 to < 10 and absolute dimension < 8 mm; and giant aneurysms are defined as *Z*_max_ ≥ 10 or absolute dimension ≥ 8 mm. Regression of CAL is defined as *Z* < 2 of all coronary arteries (LMCA, LAD, LCX, and the proximal and middle segments of the RCA) [12].

### Secondary outcome measures

1. The need for rescue therapy
2. Duration of fever (hours): from initiation of initial IVIG infusion to afebrile condition defined as axillary temperatures < 37.5°C or rectal temperature < 38°C persisting for at least 24 h
3. Occurrence of CAL at every time point at which echocardiography was performed during the study period
4. Changes in *Z* scores of LMCA, LAD, LCX, and the proximal and middle segments of the RCA throughout the study period (from admission to 12 months of illness)
5. Changes in serum CRP concentration 72 h after completion of initial IVIG infusion.
6. Frequency of AEs

### Safety

All AEs that occur during the trial period, such as death, hypertension, severe infection, allergic reactions, heart failure, and thrombosis, will be recorded and followed up until their resolution or 4 weeks after the end of the trial. The physician will judge the correlation of the events with the study drugs and record the related information, including the initiation time, duration, severity, correlation, and adjustment of treatment. Serious AEs must be immediately reported to the principal investigator.

### Adherence

First, face-to-face adherence reminder sessions will take place, including instructions about taking study medications, reinforcement of the need for regular follow-up, and notification of each assessment time point, which will also be recorded in the patient’s discharge summary. Second, the investigators will remind the patients to accept relevant examinations at every assessment time point. Third, we have a collaboration network composed of all participating centers, which may improve adherence and reduce the loss rate of follow-up. Finally, when discharged from the hospital, each patient who developed CALs will be informed of the type and frequency of cardiology assessment during the long-term follow-up (at least 3 years), which are decided by clinical experts based on risk stratification and recommendations of guidelines published in 2020 [13]. The physician will also remind the patient of the time and items for the next visit at the end of each follow-up, in hopes that these strategies will be useful for improving adherence.

The drop out criteria include discontinuation of the allocated intervention, which will be judged by the attending pediatric cardiologists due to severe side effects.

### Sample size calculation

The sample size is calculated using the SAS software (version 9.4). We assume a difference in the percentage of CAL at 1 month between the two groups of 3.3% (12.4% vs. 9.1%) [10] for the control and experimental groups, respectively. A total of 1390 cases in each group would be needed with an α of 0.05 and a power of 0.8. Given a potential dropout rate of 5% during follow-up, a total of 1500 patients in each group are planned to be enrolled.

### Statistical analysis

The primary analysis will be an intention-to-treat analysis. Per-protocol analysis will also be performed as a supportive analysis.

The generalized estimating equation model will be used to analyze the between-group difference in repeated measurements of the primary outcome (occurrence of CAL at 1 month of illness) and other secondary outcomes. Subgroup analyses will be performed based on age, gender, BMI, and type of KD diagnosis (KD or incomplete KD, defined according to the AHA diagnostic guidelines for KD, 2017 [12]) and IVIG response or not.

Descriptive statistics, such as gender, age, clinical manifestations, and subtype of KD, will be used to determine the baseline participant demographics and general status of the patients. For non-parametric data, the Mann-Whitney test will be used. Variables will be checked for normal distribution and presented as mean ± standard deviation and compared with the Student *t* test when normally distributed. For non-normally distributed variables, the data will be expressed as median ± interquartile range, and non-parametric tests will be used. Categorical variables will be expressed as number (%) and analyzed using the *χ*^2^ tests or Fisher’s exact tests when appropriate. Safety analyses will be compared with the incidence of AEs in the two groups using the *χ*^2^ test.

The confirmatory two-sided significance level will be set at 5% for all statistical tests and confidence intervals. SAS version 9.4 will be used for all analyses.

A detailed statistical analysis plan will be uploaded before the last enrolment to ClinicalTrials.gov (NCT04078568).

### Data collection, management, and monitoring

Before the recruiting starting date, all personnel involved will be trained to ensure full understanding of the research protocol and standardized measurement of the outcomes, including the internal diameters of coronary arteries. A standard case report form (CRF) has been established to record data, such as medical history, temperature records, laboratory data, echocardiography results, and auxiliary examination results.

All data will be inputted to the electronic database created by ACCESS in a timely manner. Each institution will input data offline and will upload the dataset into a cloud disk, which will be routinely monitored by a data manager from the CTU of the Children’s Hospital of Fudan University. A data monitoring committee (DMC) will be established, consisting of independent clinical experts and independent statisticians, supervising the research process according to “A proposed charter for clinical trial data monitoring committees: helping them to do their job well” [15]. Unblinded data can be accessed by members of the DMC, and the performance and safety of the trial will be reviewed weekly.

Auditing will be conducted annually, and the process will be independent from investigators.

The final closed trial dataset will be under the custody of the Children’s Hospital of Fudan University. The statisticians from the CTU of the Children’s Hospital of Fudan University will have full access to the complete, anonymized final dataset. Access to the final dataset or identifiable data by others will require written application and approval by the DMC and all study investigators. The original CRFs and consent forms will also be collected and stored securely at the Children’s Hospital of Fudan University for a period of 5 years after publication of the last paper or report from the study.

## Discussion

Since the initial report on KD by Tomosaki Kawasaki in 1967 [16], the means of reducing the occurrence of CAL has often been an issue of focus. As an adjunctive therapy for primary treatment of KD, corticosteroids are an affordable and relatively safe alternative for most patients, although their efficacy and safety need more evidence in non-Japanese populations.

According to the 2017 guidelines released by the AHA [12], the administration of a longer course of corticosteroids together with IVIG and aspirin is recommended for the treatment of high-risk patients with acute KD based on the results of the study by Kobayashi et al., but there is no consensus on the scoring systems used to identify patients at high risk for non-responsiveness to primary IVIG. Despite the extensive use of Kobayashi’s score (sensitivity and specificity of 86% and 67–68%, respectively) [17] and Egami’s score (sensitivity and specificity of 78% and 76%, respectively) [18] in screening high-risk patients in Japan, their applications are limited in other populations, including the Korean, North American, and Chinese populations, because of a lack of adequate sensitivity [19–24]. Additionally, some prediction models have been established based on the data of patients from different areas of China, such as Suzhou, Taiwan, and Beijing [21, 22, 25]. In our previous study on verifying the efficacy of the present prediction models, we found that the sensitivity and specificity were 0.272–0.799 and 0.412–0.926, respectively; none of them reached both sensitivity and specificity ≥ 0.75. These results suggest that no prediction models established based on demographic characteristics, clinical manifestations, and laboratory indexes are appropriate for clinical use to assess the risk of IVIG resistance in Chinese children with KD [24]. Nonetheless, treatment with IVIG plus corticosteroids can cause a rapid reduction of cytokine levels, which are associated with damage to the vascular endothelia [1, 26, 27]. Considering the above factors, KD patients are targeted in this trial regardless of the predicted risk of IVIG resistance.

In the previous literature, neonatal KD is rare. Hangai et al. reviewed nationwide Japanese surveys of KD from 2001 to 2012 and identified 23 neonatal cases in total, accounting for 1/5500 of patients of all ages [28]. Furthermore, there is no definite evidence for elevated risk of CAL in neonatal KD. Hangai et al. demonstrated that the risk of developing CALs was not significantly higher in neonates with KD than in older patients [28], results of which were consistent with another study involving KD patients ≤ 3 months of age [29]. Conversely, another two reports on KD emphasized the high risk of coronary artery aneurysms in patients below 6 months of age [30, 31], in which the proportion of neonates was not mentioned. Thus, the effect of excluding infants < 1 month in the final analysis may be negligible. It is believed that the use of systemic corticosteroids in newborns should be performed with great caution due to possible short- and long-term complications [32]. In consideration of safety, age was limited to 1 month and above as an inclusion criterion of this trial.

With regard to the therapeutic regimen of corticosteroids, one-off steroids and longer courses of steroids are two alternatives, and the superiority of the latter over the former has been exhibited in a meta-analysis performed by Wardle et al. [33]. In a study by Kobayashi et al., the prednisolone dose was set as 2 mg/kg per day administered over 15 days after concentrations of CRP were normalized. Consequently, patients treated with IVIG plus prednisolone defervesced more rapidly, and 2% of them had serious AEs, including elevated total cholesterol in two patients and neutropenia in one patient [9]. Based on this previous experience, the prednisolone dosage will be set as 2 mg/kg per day limited to below 60 mg, and the treatment course will be over 15 days in our trial. It is suggested that the clinical response of patients with KD may be affected by the different brands of IVIG [34], but current reports on the influence of the brands of corticosteroids and aspirin are lacking. The impact of branding on IVIG and aspirin will be balanced in the two groups in our study due to the use of blocked randomization, and there is no limit on brands of prednisolone in most previous trials [8, 9, 35–37]. The brands of the three drugs will not be restricted in this study. Additionally, we will record the brands used in every participating institution to aid in the analysis.

Although patients with KD presenting with CALs at baseline have been excluded from several trials [9, 35–37], evaluation of the effect of the additional corticosteroid to conventional initial therapy on these patients has important clinical value. It has been reported that 81% of CALs associated with KD are determined on initial echocardiography, averaging 5.4 days after onset [38, 39]. Moreover, persistent fever, one of the principal clinical features, may aggravate CALs [40]. Thus, patients with KD who develop early CALs will also be included in this study. However, patients with giant aneurysms, defined as an internal luminal diameter *Z* score ≥ 10 or absolute dimension ≥ 8 before the initial treatment and probably not regressing and adding to higher cardiac risk, such as ischemia, myocardial infarction, and ischemia-related death [41, 42], tend to accept more aggressive initial treatment, such as adjunctive infliximab. This may be beneficial to these patients by rapidly ceasing the vasculitis and preventing the deterioration of CALs [43, 44], compared to corticosteroids. In view of the balance between the clinical application and interests of patients, the highest *Z* score and absolute dimension of any coronary artery is required to be less than 10 and 8 mm before enrolment, respectively. However, the month after the onset of KD is crucial. Coronary dilatation subsiding within 1 month is considered as transient dilatation, and coronary artery sequelae are defined as coronary aneurysms persisting for longer in Japanese guidelines [39]. As indicated by a retrospective study, coronary severity 1 month after KD onset is closely related to late coronary outcomes [45]. In addition, the high persistence probability of medium and giant aneurysms that add to cardiovascular risk at 1 year has been described; however, approximately two-thirds of the acute myocardial infarction occurs within the first year of KD onset [45]. This suggests that 1 month and 1 year of illness may be key points for observing the long-term outcomes of KD. Therefore, we set the primary and final endpoint as 1 month and 1 year of illness, respectively, to assess not only whether corticosteroids could decrease cardiovascular sequelae, but also the therapeutic effect on the CALs identified before treatment.

## Trial status

Recruitment started on January 13, 2020, is ongoing at more than 20 hospitals in China and is expected to be completed in December 2021 or until a total of 3000 participants have been recruited. According to the study protocol, each patient will be followed up for 1 year. The current protocol is version 03 (January 6, 2020). At the time of manuscript submission, 1391 participants have been recruited in total, and all participants have been followed up.

## Supplementary Information

**Additional file 1.** SPIRIT checklist. A completed SPIRIT 2013 checklist.

**Additional file 2.** World Health Organization Trial Registration Data Set (Version 1.3.1).

## Data Availability

The trial results will be published in a peer-reviewed scientific paper and through oral presentations at conferences. The datasets analyzed during the current study are available from the corresponding author upon reasonable request.

## Abbreviations

AE: Adverse event
AHA: American Heart Association
CAL: Coronary artery lesion
CRF: Case report form
CRP: C-reactive protein
CTU: Clinical Trial Unit
DMC: Data monitoring committee
IVIG: Intravenous immunoglobulin
KD: Kawasaki disease
LAD: Left anterior descending artery
LCX: Left circumflex coronary artery
LMCA: Left main coronary artery
RCA: Right coronary artery
SPIRIT: Standard Protocol Items: Recommendations for Clinical Interventional Trials
