# Supplementary material for: Effects of immunoglobulin plus prednisolone in reducing coronary artery lesions in patients with Kawasaki disease: study protocol for a phase III multicenter, open-label, blinded-endpoints randomized controlled trial
Source: Trials. 2021 Dec 11;22:898. doi: 10.1186/s13063-021-05807-3 (PMC8665612; doi:10.1186/s13063-021-05807-3)
Supplement: Supplementary file 2 — Additional file 2. World Health Organization Trial Registration Data Set (Version 1.3.1). [file 13063_2021_5807_MOESM2_ESM.docx]

World Health Organization Trial Registration Data Set (Version 1.3.1)

| Items | Description |
| --- | --- |
| 1. Primary Registry and Trial Identifying Number | clinicaltrials.gov  NCT04078568 |
| 1. Date of Registration in Primary Registry | 08/16/2018 |
| 1. Secondary Identifying Numbers | NA |
| 1. Source(s) of Monetary or Material Support | Innovation Unit Project, Chinese Academy of Medical Sciences (2018RU002) |
| 1. Primary Sponsor | Chinese Academy of Medical Sciences |
| 1. Secondary Sponsor(s) | NA |
| 1. Contact for Public Queries | +86 021-64932800 |
| 1. Contact for Scientific Queries | Fang Liu, MD.  399 Wanyuan Road  Shanghai, 201102  China  Telephone: +86 021-64932800  Email: liufang@fudan.edu.cn |
| 1. Public Title | Efficacy of immunoglobulin plus prednisolone in reducing coronary artery lesions in patients with Kawasaki disease |
| 1. Scientific Title | Effects of immunoglobulin plus prednisolone in reducing coronary artery lesions in patients with Kawasaki disease: a phase III multicenter, open-label, blinded-endpoints randomized controlled trial |
| 1. Countries of Recruitment | China |
| 1. Health Condition(s) or Problem(s) Studied | Kawasaki disease |
| 1. Intervention(s) | the standard group：   1. IVIG 2g/kg once, given within 12 to 24 hours; 2. Aspirin 30 mg/kg in oral per day (given in 3 divided doses), then 3 to 5 mg/kg per day when fever subsides for 3 days and C-reactive protein (CRP) is normal. Aspirin will be continued for at least 6 weeks after onset of illness.   the standard + prednisolone group   1. IVIG 2g/kg once, given within 12 to 24 hours; 2. Aspirin 30 mg/kg in oral per day (given in 3 divided doses), then 3 to 5 mg/kg per day when fever subsides for 3 days and CRP is normal. Aspirin will be continued for at least 6 weeks after onset of illness. 3. Intravenous methylprednisolone 1.6 mg/kg per day (given in 2 divided doses) for 3 days, then changed to oral prednisolone 2 mg/kg when fever subsides for 3 days. If CRP is normal, the oral dose will be reduced every 5 days from 2 mg/kg to 1 mg/kg to 0.5 mg/kg (tapered over 15 days). Then prednisolone will be discontinued. |
| 1. Key Inclusion and Exclusion Criteria | Inclusion criteria   1. Meeting the diagnostic criteria for KD published by the American Heart Association (AHA) in 2017, including complete KD (also known as typical or classic KD) and incomplete KD (also known as atypical KD); 2. Diagnosed within 10 d of onset (including the 10th day and the 1st day of onset defined as the first day of fever); 3. Not yet treated with IVIG; 4. Age ≥ 1 month at the time of enrolment into the trial.   Exclusion criteria   1. Z score ≥ 10 or absolute dimension ≥ 8 mm of any coronary artery before enrolment; 2. History of receiving steroids or other immunosuppressive agents in the 30 days before enrolment; 3. A previous history of KD; 4. Afebrile (axillary temperature lower than 37.5^o^C for at least 24 hours) before enrolment; 5. Inability to rule out infectious diseases including sepsis, septic meningitis, peritonitis, bacterial pneumonia, varicella and influenza; 6. History of serious immune diseases such as immunodeficiency or chromosomal abnormalities; 7. Requirement for adjunctive corticosteroids or more aggressive treatments, such as biologicals, in initial therapy due to the tendency for macrophage activation syndrome or Kawasaki disease shock syndrome as judged by clinicians. |
| 1. Study Type | 1. Type of study: interventional 2. Study design including:   Method of allocation: randomized  Allocation concealment mechanism: by opaque and sealed envelopes  Sequence generation: an independent team of statisticians from the Clinical Trial Unit (CTU) of the Children’s Hospital of Fudan University will lead the generation of the randomization sequence which will be created by center using SAS (version 9.4) with block size of 4.  Masking: Single (Outcomes Assessor). Participants and physicians will not be masked to the assignment. Pediatric cardiologists who assess coronary artery lesions (CAL) by echocardiography will be masked to the allocation.  Assignment: parallel  Purpose: treatment   1. Phase: Phase 3 |
| 1. Date of First Enrollment | January 15, 2020 [actual date of enrolment of the first participant] |
| 1. Sample Size | 1. Number of participants that the trial plans to enrol in total: 3000 2. Number of participants that the trial has enrolled: 1391 |
| 1. Recruitment Status | Recruiting |
| 1. Primary Outcome(s) | Percentage of coronary artery lesions (CALs) at one month of illness [Time Frame: at one month of illness] |
| 1. Key Secondary Outcomes | 1. Percentage of the need for additional treatment   Axillary temperature (or rectal temperature) will be measured every 6 hours a day during hospitalization. Participants who have recurrent or persistent fever (axillary temperature ≥37.5°C or rectal temperature ≥38°C) after 36 hours of completion of initial IVIG infusion will be given additional treatment.  [Time Frame: from admission to discharge (about 2 weeks of illness)]   1. Duration of fever (hours) after initiation of initial IVIG infusion   Axillary temperature (or rectal temperature) will be measured every 6 hours a day during hospitalization. Participants with an axillary temperature <37.5℃ (or rectal temperature <38℃) for more than 24 hours are considered afebrile. Record the time of the initiation of IVIG infusion and the time of the body temperature first becoming normal.  [Time Frame: from initiation of initial IVIG infusion to the first record of being afebrile (defined as an axillary temperature <37.5 for more than 24 hours)]   1. Occurrence of CAL at every time point at which echocardiography was performed during the study period   [Time Frame: from admission to 12 months of illness]   1. Changes in z scores of LMCA, LAD, LCX, proximal and middle segment of RCA throughout the study period   This is a repeated measurement. The internal diameter of LMCA, LAD, LCX, proximal and middle segment of RCA will be measured by echocardiography at six time points: at enrolment, at 2 weeks, 1 month, 3 months, 6 months and 12 months of illness. Z score will be calculated based on the height, weight and coronary artery diameter（Journal of the American Society of Echocardiography, 2011, 24(1).）.  [Time Frame: from admission to 12 months of illness]   1. Change in serum C-reactive protein (CRP) concentration   CRP level is measured before initial IVIG infusion and 72 hours after completion of initial IVIG infusion.  [Time Frame: from admission to 72 hours after completion of initial IVIG infusion]   1. Number of patients with serious adverse events   This is a composite outcome, including death, hypertension, severe infection, allergic reactions, heart failure, thrombosis, etc.  [Time Frame: from admission to 12 months of illness] |
| 1. Ethics Review | Approved  Number: 2018-142  Board Name: Institutional Review Board of Children's Hospital of Fudan University  Phone: +86 021-64931913  Email: ekyykyb@163.com  Address: 399 Wanyuan Road, Shanghai, China |
| 1. Completion date | Last visit |
| 1. Summary Results | NA |
| 1. IPD sharing statement | No |
